# Sensitivity of Andes Hantavirus to Antiviral Effect of Human Saliva

**DOI:** 10.3201/eid1507.090097

**Published:** 2009-07

**Authors:** Jonas Hardestam, Åke Lundkvist, Jonas Klingström

**Affiliations:** Karolinska Institutet, Stockholm, Sweden, and Swedish Institute for Infectious Disease Control, Solna, Sweden

**Keywords:** Hantavirus, saliva, zoonoses, viruses, human-to-human transmission, Andes virus, Hantaan virus, Puumala virus, letter

**To the Editor:** Hantaviruses cause 2 severe and often fatal human diseases, hemorrhagic fever with renal syndrome (HFRS) in Eurasia and hantavirus cardiopulmonary syndrome (HCPS) in the Americas. Rodents are the natural hosts for hantaviruses that cause HFRS and HCPS, and humans are usually infected by aerosolized virus-contaminated rodent excreta (*1**,**2*). Except for Andes virus (ANDV), human-to-human transmission of hantaviruses does not seem to occur. ANDV clearly is transmitted directly from human to human (*3*), but exactly how this occurs or why other pathogenic hantaviruses are not transmitted between humans is not known.

ANDV antigen has been detected in the secretory cells of the salivary glands of humans (*4*). The risk for infection with ANDV is higher in people having sex or involved in deep kissing with an infected person than in other contacts (*5*), suggesting that transmission of ANDV needs close person-to-person contact. Therefore, one can speculate that ANDV is likely to be secreted into saliva and that saliva is involved in human-to-human transmission. Hantaviruses can be transmitted through saliva between the natural hosts (*6**,**7*), indicating that hantaviruses can withstand the antiviral effects of saliva or can interfere with production of saliva and thereby inhibit its antiviral effect.

We recently showed that saliva from Puumala hantavirus (PUUV)–infected humans contains viral RNA (*8*). This finding suggests that PUUV, and perhaps other hantaviruses, might be secreted into human saliva. However, we found no evidence of replicating virus in the saliva samples (*8**,**9*); neutralizing antibodies or salivary components may have inactivated the virus. We therefore analyzed the effect of saliva on the prototype hantavirus, Hantaan virus (HTNV). Our analysis shows that although HTNV is sensitive to the overall antiviral capacity of human saliva from healthy donors, it is insensitive to the antiviral effects of certain salivary components, i.e., histatin 5, lactoferrin, lysozyme, and secretory leukocyte protease inhibitor (*9*), which are known to have antiviral effects against other viruses.

We tested the hypotheses that ANDV might be less sensitive than HTNV and PUUV to the antiviral effect of human saliva. Saliva from healthy persons with no evidence of seropositivity against hantavirus was pooled and preincubated at different concentrations with 10,000 focus-forming units of ANDV (strain Chile-9717869), HTNV (strain 76-118), or PUUV (strain Kazan E6) for 1 hr (*9*). The virus plus saliva mixtures were then titrated on Vero E6 cells. Virus without saliva was used as a control. The medium used for dilution of saliva and virus was Hank’s balanced salt solution (Invitrogen, Paisley, UK) supplemented with 2% fetal calf serum, 2% HEPES, 100 U of penicillin/mL, and 100 µg of streptomycin/mL. Because of a cytopathic effect on the cells, we could not test saliva concentrations >50% (*9*). After incubation, titers in samples incubated with saliva were calculated and compared with titers from virus incubated without saliva.

The different hantaviruses clearly differed in their sensitivities to human saliva. At a low concentration (12.5% saliva), we observed a slight effect on HTNV, even though we saw no effect on ANDV and PUUV. ANDV was the only virus that resisted higher concentrations of saliva (25% and 50%), and an antiviral effect was clearly observed on HTNV and PUUV at these saliva concentrations (Figure).

**Figure:**
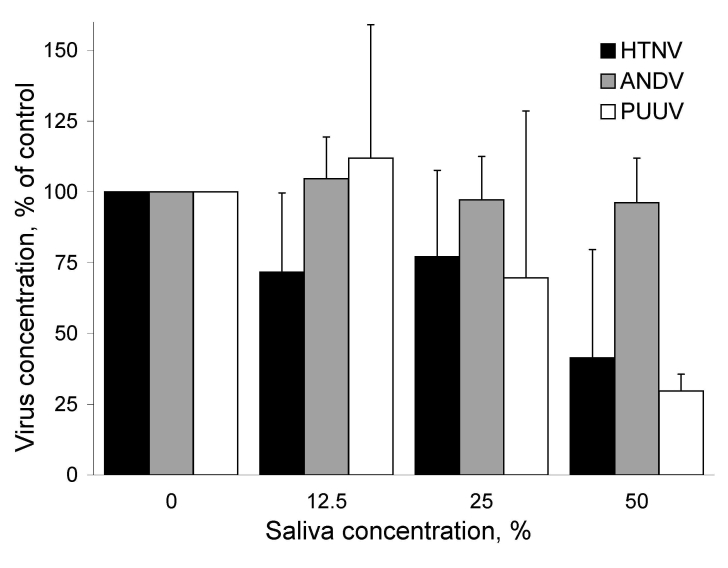
Antiviral effect of human saliva against Hantaan virus (HTNV), Andes virus (ANDV), and Puumala hantavirus (PUUV). Data represent mean + SD of 3 independent experiments.

Our finding that ANDV is less sensitive than HTNV and PUUV to the antiviral effect of human saliva might explain why ANDV, but not HTNV or PUUV, is transmitted between humans. Saliva might be the preferred route of transmission for ANDV between humans, as it is for the long-tailed rice rat (*Oligoryzomys longicaudatus*), the natural host for ANDV (*10*). However, transmission of ANDV between rodents, from rodents to humans, and between humans differs. Replicating hantaviruses have not been isolated from saliva of patients with HFRS or HCPS. In patients who have seroconverted, hantavirus-specific antibodies are likely to be present and might efficiently neutralize the virus, including ANDV. If this is the case, the interval might be short between excretion of the virus into the saliva and seroconversion, enabling the infected person to transmit hantavirus to other humans. Ferres et al. showed that in persons who developed HCPS after human-to-human transmission of ANDV, viremia preceded onset of disease and detection of ANDV-specific antibodies by up to 2 weeks (*5*). Sampling of saliva from healthy household contacts to ANDV-infected persons, with subsequent virus isolation attempts, might show whether human saliva is the mode of ANDV transfer during human-to-human transmission.
